# Urine My Heart: A Case of Aerococcal Endocarditis

**DOI:** 10.7759/cureus.14593

**Published:** 2021-04-20

**Authors:** Marium Khan, Harpreet Gill, Mudassir Khan, Vishmayaa Saravanan, Pinky Jha

**Affiliations:** 1 Internal Medicine, Medical College of Wisconsin, Milwaukee, USA; 2 Revenue Cycle, Providence St. Joseph Health, Tegria Consulting, Renton, USA

**Keywords:** aerococcus urinae, endocarditis, urinary tract infection, mass spectrometry

## Abstract

An elderly woman with previously known valvular disease presented to the emergency department due to altered mental status. In addition to obtaining an infectious work-up, a bedside echocardiogram was performed and revealed right heart strain prompting a formal echocardiogram evaluation and treatment for a possible pulmonary embolism. Initial laboratory work returned with blood and urine cultures positive for *Aerococcus urinae*. A transthoracic echocardiogram further revealed new aortic regurgitation. Given this, a transesophageal echocardiogram was completed, confirming new aortic insufficiency as well as findings of infective endocarditis. She did not undergo surgical intervention; however, she was discharged with a plan to continue intravenous antibiotics for six weeks. Although typically seen in genitourinary infections, *A. urinae* is a rare cause of infective endocarditis and is increasingly identified due to improved speciation techniques. We describe a unique presentation of invasive *A. urinae *infection to increase awareness and further research on a less commonly encountered bacteria that may present as a urinary tract infection and has the potential to cause invasive disease.

## Introduction

*Aerococcus urinae* is a rare gram-positive coccus seen in cystitis or urinary tract infections (UTI). Risk factors include older age, male sex, and pre-existing urinary tract abnormalities or benign prostatic hyperplasia (BPH) [1]. While aerococcal infections may be considered low in pathogenicity, they may result in invasive infections including bacteremia, endocarditis, peritonitis, meningitis, osteomyelitis, joint infections, or spondylodiscitis [2]. Historically, aerococcus was misidentified due to similarities with other gram-positive cocci; however, more infections are becoming properly identified due to improved mass spectrometry techniques. We describe a unique case of aerococcal endocarditis in an elderly woman with previously known valvular disease.

## Case presentation

**Figure 1:**
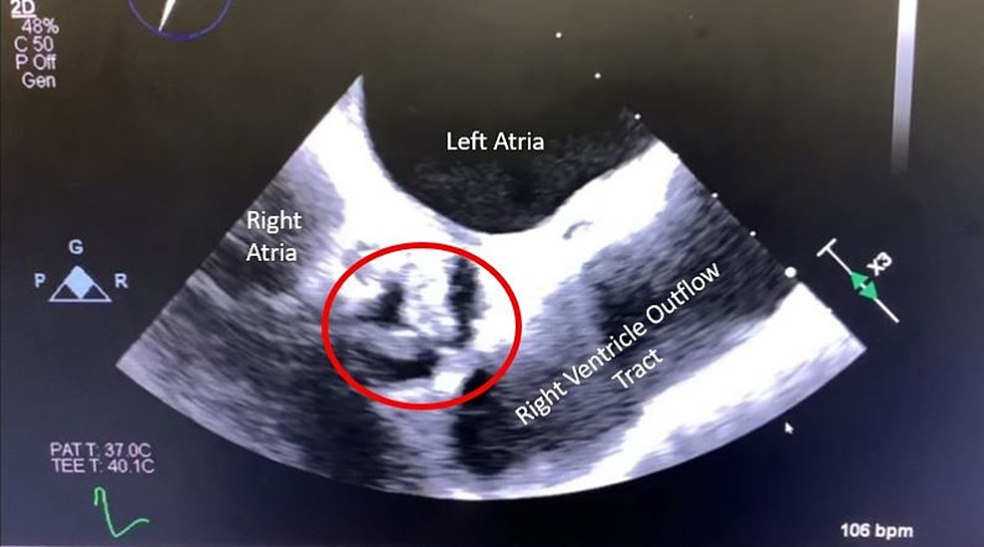
Transesophageal echocardiogram short-axis view with aortic valve vegetation circumscribed

**Figure 2:**
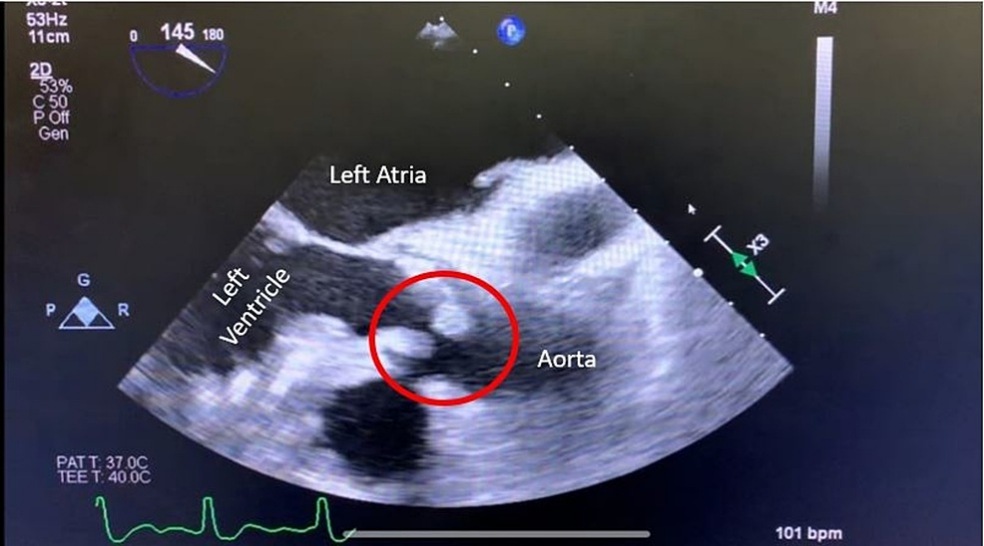
Transesophageal echocardiogram three-chamber view with aortic valve vegetation circumscribed

An 86-year-old woman with a history of atrial fibrillation, aortic stenosis, and hypertension presented to the emergency department due to altered mental status. She was lethargic, confused, and was not answering questions appropriately for the past four days. On arrival, she was afebrile, and her vitals included a pulse of 139 beats per minute, blood pressure of 110/79 mmHg, and a respiratory rate of 16 breaths per minute with saturation of 96% on room air. Physical exam was significant for a slow to respond female, orientated to self, who was able to follow commands with no focal neurological deficit. Her skin was warm and well-perfused with normal capillary refill, with no rashes or petechiae. The cardiovascular exam was significant for tachycardia, an irregular heart rhythm, and a systolic murmur heard best at the right upper sternal border. She had lower extremity pitting edema bilaterally. Her lab results were notable for elevated white blood cell count (WBC) 25.2 x 10^3^ μL (3.9-11.3 x 10^3^ μL), hemoglobin 11.0 g/dL (11.3-15.1 g/dL), platelets 58.0 x 10^3 ^μL (165-366 x 10^3^ μL), troponin 0.164 ng/mL (<0.010 ng/mL), lactic acid 2.5 mmol/L (0.5-2.0 mmol/L), and a basic chemistry panel was within normal limits. The urinalysis was remarkable for the presence of WBCs, leukocyte esterase, and bacteria with a urine culture pending. Additionally, two sets of blood cultures were obtained. Her electrocardiogram was significant for atrial fibrillation with a rapid ventricular rate (RVR). She was started on ceftriaxone for probable UTI and was admitted to the hospital for further management. Further diagnostics included lower extremity Doppler ultrasound, which revealed deep venous thrombosis (DVT). A bedside echocardiogram indicated right ventricular strain, and the N-terminal proB-type natriuretic peptide level was elevated at 8,215 pg/mL (<449 pg/mL). Due to concern for possible pulmonary embolism in the setting of known DVT, the patient was started on a continuous heparin infusion. A transthoracic echocardiogram revealed severe aortic valve calcification with new aortic insufficiency. Blood and urine cultures returned positive for *Aerococcus urinae*. A transesophageal echocardiogram further confirmed moderate to severe aortic regurgitation with findings suggestive of infective endocarditis (IE), see Figures 1, 2.

Antibiotics were expanded to include gentamicin with ceftriaxone for IE, and surgical evaluation was consulted. Given she was a high-risk surgical candidate, surgery intervention was not offered at this time unless evidence of further clinical decompensation. Additional surgical recommendations included re-evaluation after completion of antibiotic course. She was therefore discharged on intravenous antibiotics to complete a total of six weeks of treatment with ceftriaxone and two weeks of gentamicin. Soon after completion, the patient returned due to worsening thrombocytopenia that had previously improved upon discharge. On readmission, repeat labs were most notable for worsening thrombocytopenia, leukocytosis, acute kidney injury, and hyperkalemia. The patient was in atrial fibrillation with RVR and physical exam concerning for decompensated heart failure. Despite administration of broad-spectrum antibiotics and heart rate control, the patient continued to decline, and her clinical picture was consistent with cardiogenic shock. In addition to respiratory distress, the patient went into ventricular fibrillation arrest. Due to the patient's wishes to not be intubated or resuscitated, she was made comfortable and passed away.

## Discussion

The aerococci family of bacteria was first identified in 1953 and has become increasingly implicated in human disease [3]. In 1992, *A. urinae* was discovered by Aguirres and Collins in urine samples as a rare causative agent in UTIs [4]. Additional species within the aerococal genus include *A. viridans*, *A. christensenii*, *A. sanguinicola*, and *A. urinaehominis* [5,6]. *A. urinae* is an alpha-hemolytic, catalase-negative, gram-positive cocci that can appear as diplococci, tetrads, and even clusters [5]. This diverse presentation and similarity to more commonly pathologic bacteria such as *Staphylococci*, *Streptococci*, and *Enterococci *make *A. urinae* difficult to identify by culture and likely underdiagnosed. 

These qualities, in addition to some commercial labs not including tests specific for *A. urinae*, complicate making the diagnosis. In their review of 63 *A. urinae* UTI cases, Zhang et al. found that while the gold standard diagnostic test was sequencing of the 16S ribosomal RNA (rRNA), biochemical detection of leucine arylamidase, beta-glucuronidase, and pyrrolidonyl arylamidase (PYR) was an effective and more routine option [5]. In recent years, mass spectroscopy has become a powerful tool for identifying microbial organisms. Matrix-assisted laser desorption ionization-time of flight mass spectrometry (MALDI-TOF MS) in particular is well-documented in identifying *A. urinae* using non-culture techniques [1]. Conventional methods of UTI identification require 18-48 hours of culturing, while MALDI-TOF MS reduces processing time to under an hour with excellent specificity and sensitivity, further minimizing inappropriate empirical antimicrobial therapy before identification [7]. 

Other commercially available methods are unable to safely separate aerococci from other bacteria or differentiate aerococcal species from one another [2]. Senneby’s study of MALDI-TOF MS concluded a 94% median probability of correct species identification of *A. urinae* isolates while attaining a pooled specificity of 93%, which is in accordance with strong identification capabilities [2]. 

In addition to UTIs, *A. urinae* has been linked in elderly populations with comorbidities to more invasive infections [8]. In rare instances, the bacteria exit the urogenital tract hematogenously and can cause more severe, systemic complications. These presentations include necrotizing soft tissue infections, spondylodiscitis, perineal abscesses, lymphadenitis, and meningitis [1,8,9]. In a majority of cases, underlying UTI is suspected and typically has a favorable prognosis [8]. Although invasive disease is possible, literature documenting these cases is limited. For example, at the time of writing, PubMed research yielded less than 10 case reports of spondylodiscitis presentation with *A. urinae*.

IE is another systemic complication of *A. urinae* infection, as seen in the case above. In the bloodstream, *A. urinae* can seed within the endocardium in areas of disruption due to valvular disease, foreign bodies such as pacemakers, disrupted blood flow, or calcifications. In the United States, there are approximately 40-50,000 cases annually of IE [10]. Risk factors such as intravenous drug use (IVDU), intravascular catheters, immunosuppression, degenerative heart disease, diabetes, cancer, and age have largely replaced rheumatic heart disease as a cause of IE in developed countries [10,11].

*S. aureus*, a gram-positive aerobic organism, is the most recognized causative agent of IE, representing almost one-third of cases [10]. Conversely, the prevalence of *A. urinae* in IE is thought to be three in 1,000,000 [12]. While the method behind which *A. urinae* causes IE remains to be elucidated, scientific literature proposes an underlying mechanism of cellular aggregation. 

Like *S. aureus*, *A. urinae* has a strong propensity to form biofilms, which is an important step in its pathophysiology. *A. urinae* is able to produce a protective biofilm to induce platelet aggregations and ultimately form the vegetation within the endocardium [13]. A recent study by Yaban et al. used fluorescence in situ hybridization (FISH)-probe analysis to confirm two cases of in-vivo biofilm production by *A. urinae* [14]. Biofilm production by *A. urinae* allows for the bacteria to settle not only within the urinary tract system but as well as within the endocardium, including heart valves. Once the bacteria are able to adhere onto these surfaces, it aggregates along with platelets and other inflammatory response cells increasing the risk of vegetation formation [6].

*A. urinae* incidence increases with age and tends to present more in men than women [1,5,13]. A 2018 review by Yabes et al. found only 43 cases in the literature with a mean age of 73 and in which over 80% were men (n = 36) [1]. Presence of comorbid urogenital abnormalities is associated with a higher risk of *A. urinae* infection. Other risk factors include frequent UTIs, prostatitis, BPH, or an indwelling Foley catheter [1]. The increased prevalence in males may be linked to development of prostatic hyperplasia and incidence of bacterial prostatitis [15,16]. Although *Enterobacteriaceae* are the most common causative organisms in both acute bacterial prostatitis (ABP) and chronic bacterial prostatitis (CBP), gram-positive organisms are found in both types and more commonly in CBP [16]. Despite the increased risk of *A. urinae* infection among males and those with pre-existing urogenital abnormalities, our case patient presented without the typical urogenital risk factors. However, she did have previously known valvular disease and was 86 years old, likely predisposing her to IE.

While there are no specific recommendations, *A. urinae* is sensitive to beta-lactam medications and resistant to sulfonamides and most notably fluoroquinolones, which are commonly used to treat bacterial prostatitis [1,16,17]. In particular, *A. urinae* IE responds well to beta-lactams and aminoglycosides. In all 43 cases reviewed by Yabes et al., this was the mainstay of the treatment [1]. Vancomycin therapy can be considered for more severe cases or individuals allergic to penicillin [1].

Synergistic behavior with beta-lactams and aminoglycosides can last anywhere from 10 days to six weeks [1]. Despite the empirical success of beta-lactams and aminoglycosides for treatment options, the approach is not universal. One of the greatest variables limiting prolonged use of aminoglycosides includes toxicity such as nephrotoxicity and ototoxicity [18]. Furthermore, in one study, *A. urinae* isolates failed to show synergistic effects of beta-lactam gentamicin therapy approximately 50% of the time [19]. Given that possible outcomes range from synergistic effects to drug resistance and fatal consequences, timely and accurate identification of *A. urinae* using improved techniques is of vital importance in appropriate treatment strategy. In addition to antibiotic therapy, evaluation for removal of the vegetation should be considered by expert cardiothoracic surgery consultation.

## Conclusions

Although presentation of *A. urinae *is uncommon, clinicians should be aware of the possibility of invasive *A. urinae* infections such as IE as seen in our case patient. Due to improved diagnostic techniques in microbiology identification, more cases of *A. urinae* are being identified allowing for prompt and proper treatment. This case report adds to the limited literature on invasive *A. urinae* infections, including IE. Further research must be conducted in order to develop guidelines on treatment and management of *A. urinae* infections. As clinicians, we must continue to have a high index of suspicion for more invasive disease when identifying *A. urinae* infections.
